# A cluster-randomized controlled study to evaluate a team coaching concept for improving teamwork and patient-centeredness in rehabilitation teams

**DOI:** 10.1371/journal.pone.0180171

**Published:** 2017-07-13

**Authors:** Mirjam Körner, Leonie Luzay, Anne Plewnia, Sonja Becker, Manfred Rundel, Linda Zimmermann, Christian Müller

**Affiliations:** 1 Medical Psychology and Medical Sociology, Medical Faculty, Albert-Ludwigs-University, Freiburg, Germany; 2 Celenus-Kliniken GmbH, Offenburg, Germany; 3 Moving-Concept, Freiburg, Germany; 4 Saarland University of Cooperative Education in Health Care and Welfare, Saarbrücken, Germany; Universite de Bretagne Occidentale, FRANCE

## Abstract

**Purpose:**

Although the relevance of interprofessional teamwork in the delivery of patient-centered care is well known, there is a lack of interventions for improving team interaction in the context of rehabilitation in Germany. The aim of the present study is to evaluate whether a specially developed team coaching concept (TCC) could improve both teamwork and patient-centeredness.

**Method:**

A multicenter, cluster-randomized controlled intervention study was conducted with both staff and patient questionnaires. Data was collected at ten German rehabilitation clinics (five clusters) of different indication fields before (t1) and after (t2) the intervention. Intervention clinics received the TCC, while control clinics did not receive any treatment. Staff questionnaires were used to measure internal participation and other aspects of teamwork, such as team organization, while patient questionnaires assessed patient-centeredness. A multivariate analysis of variance was applied for data analysis.

**Results:**

In order to analyze the effect of TCC on internal participation and teamwork, 305 questionnaires were included for t1 and 213 for t2 in the staff survey. In the patient survey, 523 questionnaires were included for t1 and 545 for t2. The TCC improved team organization, willingness to accept responsibility and knowledge integration according to staff, with small effect sizes (univariate: *η*^*2*^*=*.010–.017), whereas other parameters including internal participation, team leadership and cohesion did not improve due to the intervention. The patient survey did not show any improvements on the assessed dimensions.

**Conclusion:**

The TCC improved dimensions that were addressed directly by the approach and were linked to the clinics’ needs, such as restructured team meetings and better exchange of information. The TCC can be used to improve team organization, willingness to accept responsibility, and knowledge integration in rehabilitation practice, but some further evaluation is needed to understand contextual factors and processes regarding the implementation of the intervention.

## Introduction

Interprofessional teamwork is becoming more and more significant based on current developments, for instance the discussion of quality and safety of care, the focus on patient-centered care, shifting demographics and an increase in chronic illnesses, patient empowerment and participation linked with rising consumerism and increasing costs of care [1]. Interprofessional teamwork in health care is defined as a collaborative interaction among at least two different health care professionals with different abilities and fields of activities to solve a common task and reach a common goal [2]. Key dimensions of interprofessional teamwork are clear goals, shared team identity, shared commitment, clear team roles and responsibilities, interdependence between team members and integration of different work practices [1]. Additional important elements include good communication, understanding of the other persons’ roles, the development of joint protocols, training and work practices, and regular and effective team meetings [1, 3, 4]. Teamwork in healthcare is proven to have benefits for patients, for example enhanced satisfaction, acceptance of treatment and improved health outcomes, as well as for team members, such as enhanced job satisfaction, greater role clarity and enhanced well-being [5].

Besides interprofessional teamwork, patient-centeredness is another important concept in modern healthcare and is emphasized as an important quality and outcome criterion [6–8]. In contrast to traditional health care models, the provider’s focus in patient-centered care is “on the patient versus the health concern or problem” [9]. There are several concepts and models of patient-centeredness (e.g. [8, 10–13]). Some of them focus mainly on the patient-physician/professional interaction [12, 14, 15], whereas others are broader and also consider organizational and structural aspects [6, 8, 13, 16]. Concerning these organizational and structural aspects, interprofessional teamwork and coordination play an important role [17]. Scholl et al [13] conducted a review in order to postulate an integrated model of patient-centeredness, which includes the dimensions patient as individual, patient participation in the treatment process, patient information, healthcare professional-patient communication and patient empowerment. In addition to these points, the model of integrated patient-centeredness developed by Körner et al. [8] includes internal and external participation as two aspects of patient centeredness. While external participation emphasizes patient-provider communication, coordination, and cooperation such as shared decision making, internal participation, which will be the focus of this study, focuses on teamwork. Research findings show that interprofessional teamwork is a main predictor of patient-centeredness [3]; therefore interprofessional teamwork is regarded as a key component of patient-centered treatment in healthcare. This is particularly important in the rehabilitation sector, where many different health professionals work together in interprofessional teams in order to provide high quality and safe treatment for patients with chronic conditions [1, 2, 18].

Interventions aiming to improve patient-centered care often focus on external participation or rather patient-professional interaction, e.g. shared decision-making [3, 8] or health coaching [19, 20]. However, it could also be shown that team interventions in health care have a positive impact on interprofessional teamwork [1, 21, 22]. Internationally, several team interventions are available for the health care sector [1, 4, 23]; they include team trainings, integrated care pathways, case management, feedback sessions or changes in team composition, such as the establishment of a new position in the team. It has been concluded that “the optimal approach is the implementation of a combination of interventions, with adaptations to fit unique clinical settings and local culture” [23]. A team coaching intervention can be defined as “collaborative, individualized, solution focused, results orientated, systematic, stretching, (and it) fosters self-directed learning”[24]. Team coaching can therefore be described as enabling team members to make use of their collective resources [25] rather than deliver proposed solutions. The effectiveness of coaching in health care teams has hardly been evaluated so far; simultaneously, the use and differentiation of terminology such as team training, team building and team coaching is blurred in the field of interventions in healthcare. One study by Klein and colleagues [26] investigates the effectiveness of team building actions which ought to consist “of four components: 1) goal-setting, 2) interpersonal relations, 3) role clarification, and 4) problem solving”. It could be shown that such team building interventions had a moderate effect on team outcomes, especially on affective outcomes (e.g. trust, attitude) and process outcomes (e.g. communication, coordination), and the component goal setting accounted for the most variance in team functioning (14%). The effectiveness and implementation of healthcare team training have been examined in several reviews [21–23, 27–30], but they all focused on interventions in acute care and post-acute care settings. The results are mixed and vary among types of intervention and health care settings. One systematic review in chronic care found 14 intervention studies. Most of them combined different actions for improving the teams and were very heterogeneous in content and complexity. For all interventions except one, positive effects were described [4]. Nonetheless, no studies on a TCC for interprofessional teams could be found. In summary, it must be emphasized that comprehensive models of patient-centeredness [8, 13] consider (interprofessional) teamwork as an enabler, but there are currently no interventions for improving team interaction and in consequence patient-centeredness at rehabilitation clinics in Germany.

### Aim of the study

The aim of the present study was to evaluate the below-described TCC. The research questions were as follows:

Can the TCC improve a) teamwork and b) patient-centeredness?

Based on the model of integrated patient-centeredness [8], we expected that the TCC can improve both aspects. On the basis of research findings suggesting that team interventions in health care have a positive impact on teamwork [1, 4, 23], the first hypothesis is *that the TCC will improve interprofessional teamwork in medical rehabilitation*. Furthermore, previous research has shown that interprofessional teamwork is a main predictor of patient-centeredness [18]. Therefore, it can be expected *that the TCC will enhance patient-centeredness* (hypothesis 2).

## Methods

A multicenter, cluster randomized, controlled intervention study was used for evaluation. Data was collected during two data collection periods (pre-intervention from June to September 2013, post-intervention six months after the implementation of the team intervention) by means of patient and staff surveys. After the first data collection period, the team intervention approach was implemented at five intervention clinics, whereas the control clinics received no intervention. The study was approved by the Ethics Committee of the University of Freiburg (official approval number: 190/12). A positive ethics committee vote is available.

### Team coaching concept (TCC)

The TCC was developed for medical rehabilitation based on a systematic literature search on team development [4] and a qualitative pilot study including interviews with executives, group interviews with team members as well as focus groups with patients. The pilot study shows that the wishes and requests concerning team coaching varied widely among the clinics [31]. Therefore a standardized training program is not possible. However, the TCC is standardized in its process but not in content, meaning that the individual needs and requests of each clinic can be taken into consideration. The TCC [32] concentrates on working processes, team organization, distribution of roles and responsibilities and optimization of communication rather than focusing on changes in individual team members. The aim is to enhance teamwork and team performance. Methodologically we combined solution-focused, task-related and systemic team development approaches into one concept. The process includes the following four distinct, sequential phases:

1. Identification of the expectations for team coaching (need-specific)
2. Definition of the coaching goals (task-related)
3. Development of the solution (solution-focused)
4. Maintenance of the solution (systemic)

**Fig 1:**
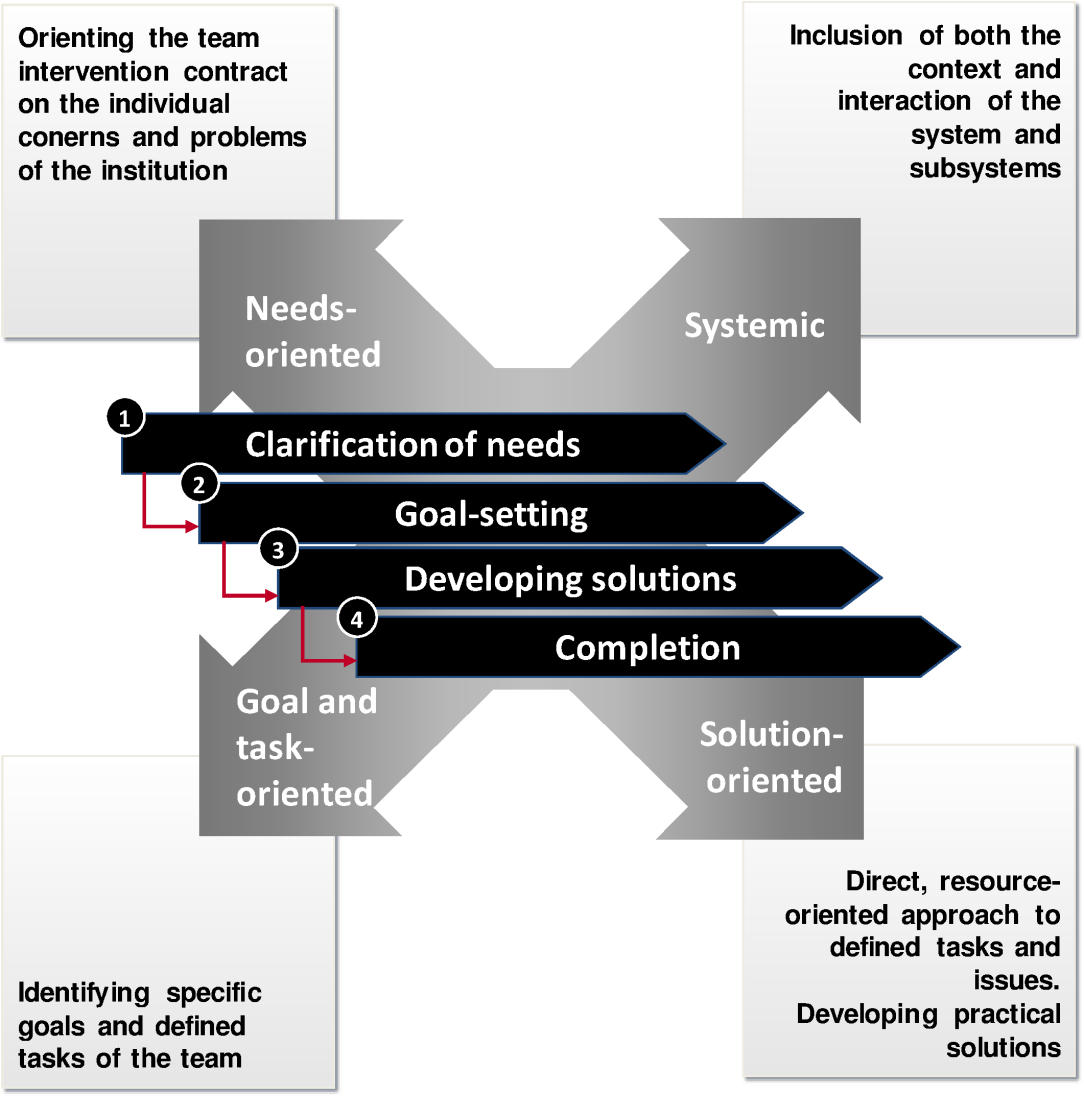
Team coaching concept (TCC).

The aim of the first phase is to identify the clinic’s expectations (clarification of the contract) for TCC and to specify the tasks of the interprofessional team in collaboration with the medical director, the administration manager and optimally the leader of the team in which the intervention should be implemented. It is checked whether the expectations can be met via the method of TCC. A request could be to “optimize the selection of patients that are discussed in the interprofessional team meeting” and to “facilitate the exchange of information regarding the patients among the different professional groups”. In the second phase, the tasks and objectives defined by the executives are discussed within the team to establish a consensus between staff and executives concerning the goals and tasks of the team (target state). During this process, the goal should be clearly and measurably specified and conceptualized (e.g. “all the information needed to reach the rehabilitation goal is accessible to every team member”). To determine the current state, every team member is asked to rate to what extent the goal has already been reached at this point (current state). In the third phase, ideas are collected on how to close the gap between the current and target state. These ideas are prioritized and discussed with respect to their practicability and benefits, and precise steps and responsibilities are blueprinted for better implementation. The continuous process is oriented towards resources and solutions. At the end of the training, in phase four, a procedure is outlined for maintaining the results within the organization (system), and responsibilities for the future are agreed upon. The essential points of the TCC are described more precisely in a manual available in German and English, which can be requested from the first author of this article. The German version is available online [33]. The concept was implemented at five rehabilitation clinics (one interprofessional team per clinic), which were then compared to five control clinics that had received no intervention. At each clinic, two trainers were responsible for the sessions. The trainers were part of the research team, were skilled in systemic coaching and had been involved in the development of the TCC. The number of sessions and time span between these sessions differed between the clinics, ranging from 1 to 6 sessions within the time span of 1 to 15 months, according to goals and processes. In total, 71 participants took part in the intervention. Fig 1 summarizes the TCC.

### Recruitment

The principles of the team development approach require implementation on a group level (teams), but outcome criteria were assessed on an individual level. For this reason, and due to the fact that the study was conducted at clinics of different specializations, cluster randomization was necessary. We intended to use five clusters. For inclusion in clusters, clinics had to be rehabilitation clinics in Germany, and the approval of clinic management had to be available. One hundred fourteen clinics in southwest Germany were informed about the project and offered the option of participating. The clinics were extracted from a web-based database (www.rehakliniken.de). Out of these, 24 clinics were generally interested in participation. After contact was initiated and further information provided, ten rehabilitation clinics agreed to participate. These ten clinics (clusters) were placed in pairs that were matched as closely as possible in terms of their specialization (orthopedics, cardiology, oncology and neurology) and size (80–310 beds). Randomization was insured by writing the names of the clinics down and blindly drawing them from a box in order to allocate them to the intervention group (the matched clinic with the same indication field was accordingly assigned to the control group). Each clinic determined a contact person responsible for the study process, and all questionnaires were sent to this person, who distributed them at the clinic. The number of questionnaires they received depended on the information they provided on clinic size. Patients were not aware of which group they belonged to, whereas the study coordinator and staff were.

At two data collection times (time 1 (t1) and 6 months after intervention=time 2 (t2)), all healthcare professionals working at the clinic were asked to complete the survey anonymously, with participation being optional. Regarding patients, physicians were asked to hand out the questionnaire to every patient they treated until all questionnaires were handed out. Therefore, the patient sample was not dependent, whereas the staff sample was at least partly dependent (but not completely, due to absence, leave or dropout). To be included in the study, staff members had to be health care professionals, work at rehabilitation clinics, have patient contact, have been members of the rehabilitation team for over 1 year, be actively practicing, be over 18 years of age and have sufficient German language abilities. All members of staff who met these criteria were asked to participate in the survey. This was done because a dissemination effect in multi-team systems in rehabilitation was expected, meaning that the achieved effects in the trained team would spread to other teams in the clinic [32]. Inclusion criteria for patients were suffering from chronic disease(s), receiving inpatient rehabilitation, being over 18 years of age, having sufficient German language abilities, exhibiting no major cognitive impairments and having signed the informed consent form. Table 1 specifies case numbers, indication fields and cluster allocations of each clinic.

**Table 1:** Clinic characteristics.

					Staff sample						Patient sample					
ID	group	Beds *n*	Indication specification	Cluster	Pre-Intervention			Post-Intervention			Pre-Intervention			Post-Intervention		
					*n*	*rr*	*rr(%)*	*n*	*rr*	*rr(%)*	*n*	*rr*	*rr(%)*	*n*	*rr*	*rr(%)*
1	IG	140	oncology	I	41	17	41%	39	19	49%	100	84	84%	100	78	78%
7	CG	152	oncology	I	100	44	44%	44	20	45%	25	22	88%	25	21	84%
2	IG	188	neurology	II	144	46	32%	103	34	33%	90	65	72%	44	38	86%
8	CG	215	neurology	II	121	55	45%	78	29	37%	-	-	-	-	-	-
3	IG	229	orthopaedics	III	93	36	39%	77	24	31%	140	99	71%	140	88	63%
9	CG	260	orthopaedics	III	140	37	26%	71	24	34%	77	61	79%	110	78	71%
4	IG	310	orthopaedics/cardiology	IV	109	40	37%	108	28	26%	100	73	73%	143	109	76%
6	CG	80	orthopaedics/cardiology	IV	37	16	43%	31	16	52%	83	53	64%	80	58	73%
5	IG	124	orthopaedics	V	70	11	16%	47	18	38%	135	39	29%	81	81	100%
10	CG	106	orthopaedics	V	35	15	43%	35	14	40%	100	43	43%	45	16	36%

ID: Clinic Identification number of clinics, n: questionnaires sent to the clinic, rr: total number of questionnaires received back, rr%: response rate (percentage of questionnaires received back among distributed questionnaires), IG: Intervention Group, CG: Control Group

**Fig 2:**
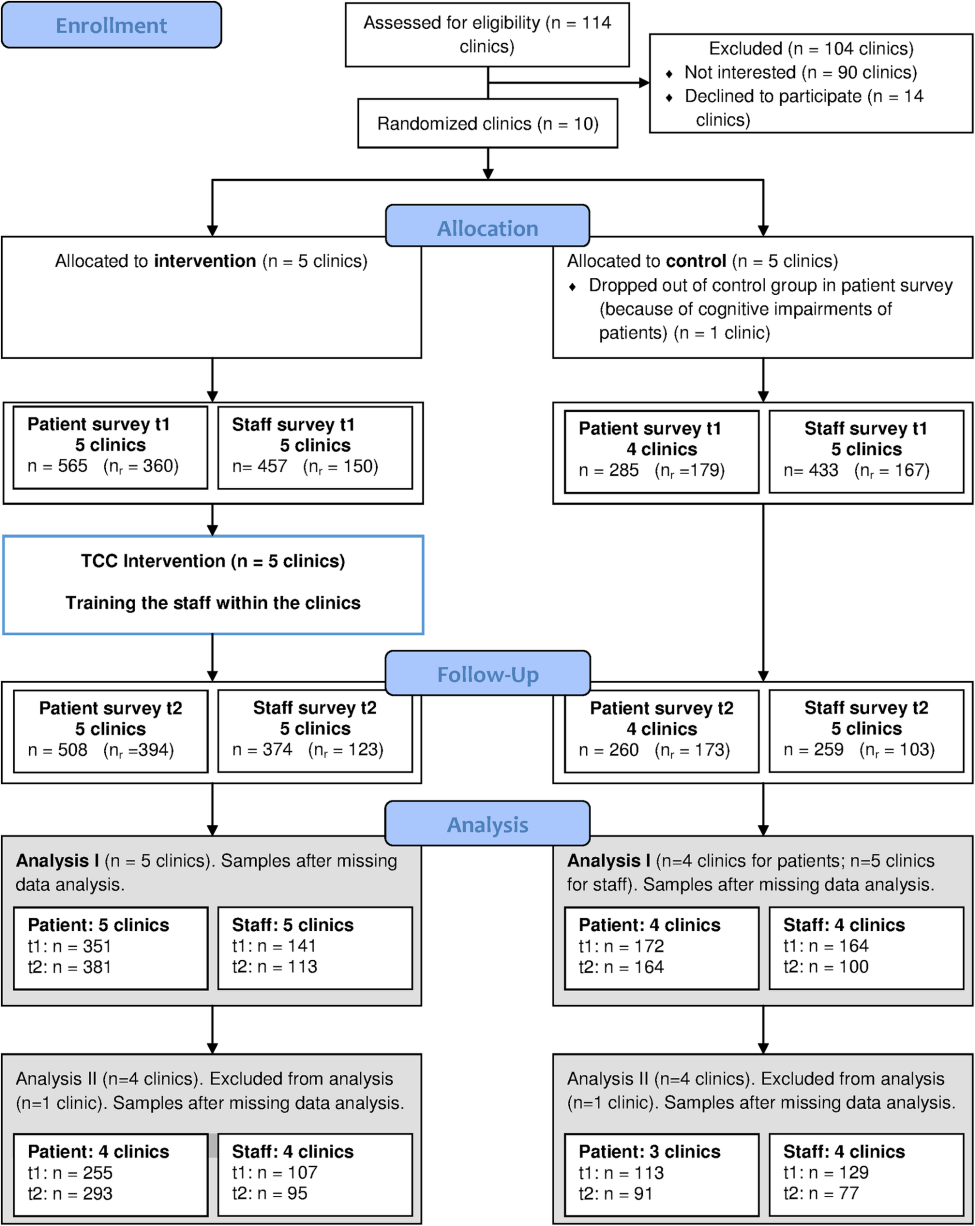
Flowchart study process. *Legend*: n=number of questionnaires sent to clinics, (nr)=number of questionnaires returned, t1=pre-intervention, t2=post intervention, TCC=Team Coaching Concept.

At one neurological clinic (clinic 8), patients could not fill out the questionnaires due to cognitive impairments. This clinic treats patients with very severe brain injuries (early stage of rehabilitation, which is phase B according to the German national association for rehabilitation), whereas the treatment focus of its matched control clinic is on occupational rehabilitation for less impaired patients (phases C and D). Ultimately, only nine clinics took part in the patient survey (see Fig 2).

### Assessment

Whereas staff questionnaires (see S1 Quest) were used to measure internal participation and other aspects of teamwork like team organization and leadership, patient questionnaires (see S2 Quest) aimed to assess external participation for patient-centeredness [8, 16]. The following instruments were used:

#### Staff questionnaire

The *Internal Participation Scale (IPS)* is based on the model of patient-centeredness [16, 34] and defines internal participation as interprofessional, patient-centered teamwork, including processes like communication, cooperation, coordination, climate, agreement and respect. The items of the scale can be rated on a four-point Likert scale, ranging from 1 to 4 (1=does not apply at all, 2=does not generally apply, 3=generally applies, 4=fully applies), with the additional option “I can’t judge this.” Internal consistency can be considered as good, with Cronbach’s alpha equaling .87 for the staff sample [34].

For assessing team leadership and team organization, two scales of the *TeamPuls* questionnaire (eight items each) were used. The ratings were also based on a four-point Likert scale from 1 (does not apply at all) to 4 (fully applies) [35]. The reliability of the scales can be considered good (team leadership α=.91, team organization α=.80).

The *Questionnaire on Teamwork (FAT)* [36] was used to measure four further aspects of teamwork. The scale on “structure orientation” includes the subscales “objective orientation” and “task accomplishment.” The scale on “person orientation” is composed of the subscales on “cohesion” and “willingness to accept responsibility.” The subscales build upon each other. The questionnaire consists of 24 items. The items are bipolar, for instance “The objectives of the team are clear” versus “The objectives of the team are unclear.” The reliability of structure orientation is α=.83 and person orientation α=.89.

The modified *“Scale of knowledge integration problems”(KIP Scale;* in German: *WIP-Skala)* [37] was applied to assess knowledge integration in the interprofessional team. The first item of the original questionnaire (“The team members are not prepared to consider other points of view“) was eliminated in order to shorten the questionnaire and to adapt it to the present research context. This left a total of seven Likert-scaled items. The scales ranged from 0 (does not apply at all) to 4 (fully applies). Internal consistency can be evaluated as good (Cronbach´s α=.86) [37].

#### Patient questionnaire

In the *patient survey*, the *Client-Centered Rehabilitation Questionnaire (CCRQ)* by Cott, Teare, McGilton and Leneker [11] was translated into German and underwent confirmatory testing [38]. In the original version, it consists of 33 items that are matched to seven scales: participation in decision-making and goal-setting, client-centered education, client evaluation of outcomes, family involvement, emotional support, physical comfort, and coordination and continuity. The confirmatory factor analysis with the patient sample from the first data collection period did not allow the replication of the seven scales. Instead a three-factor structure emerged. The revised and validated short-version CCRQ-15 surveys [38] exhibits 15 items in the following three dimensions of patient-centeredness: decision-making/communication *(CCRQ-scale 1)*, self-management/empowerment *(CCRQ-scale 2)*, and psychosocial well-being *(CCRQ-scale 3)*. The four to six items on three scales are rated using a five-point Likert Scale (1=strongly disagree to 5=strongly agree). The response option "does not apply" (= 0) was also available. High item scores (and high subscale scores) stand for higher perceived patient-centeredness. The internal consistency of the scales results in Cronbach’s α=.83–.87.

### Data analysis

Data quality was controlled by means of double data entry of random samples and verification of plausibility. Missing data analysis was performed, and questionnaires with more than 30% missing values were excluded [39]. The extent of within-cluster similarity for the end points (dependent variables) as an important design feature of a cluster-randomized controlled study was tested by calculating the intraclass correlation coefficient (ICC) for all data collection periods (for staff and patient samples). Hierarchical linear modelling only allows a solid estimation of level-two effects if the study sample consists of at least 30 level-two units (rehabilitation clinics) and the ICC is bigger than 0.1 [40, 41]. Since our study comprised not more than 10 rehabilitation clinics and the ICCs do not meet the criteria, an analysis that takes into account the two-level structure could not be applied. Therefore, data collected on an individual level was aggregated to a group level (intervention vs. control group) for each data collection period (t1 and t2). On a cluster-level, only descriptive analysis was done. For the comparison of the intervention and control groups, pre- and post-intervention multivariate analysis of variance (MANOVA) was performed to investigate differences in teamwork variables (internal participation, team organization and team leadership, objective orientation, task accomplishment, cohesion, willingness to accept responsibility, problems with knowledge integration). Requirements for computation of a MANOVA were checked but not met, but since no established non-parametric methods are available for this research question, the MANOVA was still calculated. For the patient survey, a MANOVA was equally performed to analyze time and group differences in decision-making/communication (CCRQ-scale 1), self-management/empowerment (CCRQ-scale 2), and psychosocial well-being (CCRQ-scale 3). The extent of differences between the groups was measured using partial eta-squared (*η*^*2*^) as effect size, categorized as follows: *η*^*2*^=0.01(small); *η*^*2*^=0.06 (medium); *η*^*2*^=0.14 (high) [42]. Additionally, individual ANOVA analyses were carried out to detect for which individual teamwork variables effects of the team intervention could be shown. Data was analyzed using IBM Statistics SPSS (Version 22) for Windows (see S1 and S2 SPSS). The alpha level was set to .05.

Since persisting conflicts at one clinic had a negative effect on team development and could not be resolved by the method, the effectiveness of and satisfaction with the concept were evaluated negatively in the process evaluation of this clinic [43]. Thus this clinic and its matched control clinic (clinics 3 and 9) were excluded in a second data analysis to be able to estimate the effect of the intervention on teamwork under the condition that the approach is accepted by the interprofessional team. For reduced sample sizes, see Fig 2.

## Results

### Sample of healthcare professionals

At t1 and t2, 890 and 633 questionnaires were distributed to staff, and 317 and 226 questionnaires were completed. This equaled a response rate of 37% and 36%, respectively (for clinic-specific response rates see Table 1).

Table 2 illustrates the healthcare professional samples for the data collection periods. Regarding age, gender, occupational groups and percentage of working hours, samples were similar for both periods of data collection. The majority of professionals was older than 40 years, female and worked at the respective clinic 100% of their working hours. Physical therapists were the biggest occupational group. Of the 113 participants analyzed at time t2 at the intervention clinics, 56 took part in the intervention.

**Table 2:** Distribution of healthcare professionals for the pre- and post-intervention periods.

		Intervention group				Control group			
		t1 *(n*=141)		t2 *(n*=113)		t1 *(n*=164)		t2 *(n*=100)	
		*n*	*%*	*n*	*%*	*n*	*%*	*n*	*%*
**Age group**	< 30 years	17	12.1	21	18.6	19	11.6	19	19.0
	30 to 39 years	18	12.8	13	11.5	22	13.4	18	18.0
	40 to 49 years	43	30.5	30	26.5	62	37.8	24	24.0
	50 to 59 years	49	34.8	39	34.5	48	29.3	28	28.0
	> 59 years	7	5.0	5	4.4	11	6.7	11	11.0
	Missing	7	5.0	5	4.4	2	1.2	0	0.0
**Gender **	Female	96	68.1	70	61.9	111	67.7	68	68.0
	Male	38	27.0	38	33.6	49	29.9	31	31.0
	Missing	7	5.0	5	4.4	4	2.4	1	1.0
**Occupational group**	Physician	27	19.1	21	18.6	27	16.5	22	22.0
	Nursing staff	33	23.4	27	23.9	48	29.3	23	23.0
	Physical therapist	44	31.2	34	30.1	46	28.0	27	27.0
	Psychosocial therapist	16	11.3	11	9.7	12	7.3	9	9.0
	Other	13	9.2	12	10.6	21	12.8	14	14.0
	Missing	8	5.7	8	7.1	10	6.1	5	5.0
**Percentage of working hours at the clinic **	< 50%	6	4.3	6	5.3	13	7.9	7	7.0
	≥ 50% < 75%	26	18.4	15	13.3	35	21.3	17	17.0
	≥ 75% < 100%	24	17.0	19	16.8	24	14.6	13	13.0
	100%	80	56.7	70	61.9	91	55.5	63	63.0
	Missing	5	3.5	3	2.7	1	0.6	0	0.0
**Job tenure**	0 to 5 years	49	34.8	47	41.6	46	28.0	38	38.0
	6 to 10 years	20	14.2	18	15.9	28	17.1	18	18.0
	11 to 15 years	31	22.0	15	13.3	31	18.9	10	10.0
	> 15 years	37	26.2	30	26.5	57	34.8	34	34.0
	Missing	4	2.8	3	2.7	2	1.2	0	0.0

t1: period of data collection before intervention; t2: period of data collection six months after intervention

### Patient sample

In total, 990 patient questionnaires were sent to nine clinics at t1, of which 850 were handed out. The questionnaires were completed by 539 patients, which led to a response rate of 63%. Out of the 768 patients asked at t2 (*n*=940 sent out), 567 filled out the questionnaire, resulting in a response rate of 74%. Clinic-specific response rates are displayed in Table 1. The difference in questionnaires distributed in the patient sample was due to the distribution process in the clinics and due to the fact that not the same cohort of patients was examined in the pre and post survey.

Overall, more women than men participated in the patient survey (see Table 3). Most of the patients were married, and most indicated a lower education level. The majority was no longer employed. This is compatible with the high average age of the sample (*M*=62.73, *SD*=12.80). In terms of specialization, it is important to note the large percentage of orthopedic (and rheumatologic) indications. The onset of illness was between six months and three years ago for most of the patients, and another large percentage indicated an onset more than six years ago.

**Table 3:** Patient sample for t1 and t2.

		Intervention group				Control group			
		t1*(n* = 351)		t2 *(n = *381)		t1 *(n* = 172)		t2 *(n* = 164)	
		*n*	*%*	*n*	*%*	*n*	*%*	*n*	*%*
**Age**	< 30 years	9	2.6	2	.5	3	1.7	0	0.0
	30 to 39 years	16	4.6	8	2.1	5	2.9	2	1.2
	40 to 49 years	55	15.7	49	12.9	12	7.0	15	9.1
	50 to 59 years	116	33.0	126	33.1	25	14.5	32	19.5
	> 59 years	155	44.2	194	50.9	127	73.8	114	69.5
	Missing	0	0.0	2	0.5	0	0.0	1	0.6
**Gender**	Female	216	61.5	223	58.5	98	57.0	86	52.4
	Male	131	37.3	153	40.2	70	40.7	76	46.3
	Missing	4	1.1	5	1.3	4	2.3	2	1.2
**Family status**	Single	51	14.5	36	9.4	18	10.5	10	6.1
	Divorced	60	17.1	43	11.3	5	2.9	21	12.8
	Married	209	59.5	260	68.2	124	72.1	109	66.5
	Widowed	28	8.0	34	8.9	17	9.9	24	14.6
	Missing	3	.9	8	2.1	8	4.7	0	0.0
**Education**	No school leaving qualification	1	.3	4	1.0	1	.6	3	1.8
	Elementary/ secondary school qualification	133	37.9	136	35.7	78	45.3	70	42.7
	Middle school leaving qualification	117	33.3	117	30.7	41	23.8	46	28.0
	Advanced technical college certificate	34	9.7	44	11.5	17	9.9	9	5.5
	General university entrance qualification	55	15.7	65	17.1	31	18.0	26	15.9
	Other school leaving qualification	8	2.3	7	1.8	1	.6	8	4.9
	Missing	3	.9	8	2.1	3	1.7	2	1.2
**Employment**	Yes	185	52.7	194	50.9	48	27.9	65	39.6
	No	161	45.9	179	47.0	117	68.0	98	59.8
	Missing	5	1.4	8	2.1	7	4.1	1	0.6
**Indication**	Orthopaedic/ rheumatologic	181	51.6	216	56.7	140	81.4	125	76.2
	Cardiologic	2	.6	37	9.7	0	0.0	1	.6
	Neurologic	67	19.1	42	11.0	2	1.2	6	3.7
	Oncologic	77	21.9	66	17.3	10	5.8	13	7.9
	Other	20	5.7	1	.3	0	0.0	0	0.0
	Missing	4	1.1	19	5.0	20	11.6	19	11.6
**Duration of illness**	< 3 months	59	16.8	69	18.1	18	10.5	24	14.6
	3 to 6 months	53	15.1	52	13.6	19	11.0	14	8.5
	6 to 12 months	74	21.1	75	19.7	25	14.5	29	17.7
	1 to 3 years	69	19.7	78	20.5	36	20.9	39	23.8
	3 to 6 years	35	10.0	24	6.3	23	13.4	23	14.0
	> 6 years	58	16.5	73	19.2	45	26.2	32	19.5
	Missing	3	.9	10	2.6	6	3.5	3	1.8

t1: period of data collection before intervention; t2: period of data collection six months after intervention

### Intraclass correlation coefficient

The ICCs were below 0.1 for the dependent variables in all data collection periods in the staff sample and for most of the variables in the patient sample (see Table 4) [40, 41] so that the aggregation of data of different clinics into groups (intervention- and control group) was justified.

**Table 4:** ICC for independent variables for staff and patients.

	t1	t2
**Staff**		
Internal participation (IPS)	0.075	0.038
Team leadership (TeamPuls)	0.039	-0.012
Team organization (TeamPuls)	0.049	-0.010
Knowledge integration problems (WIP)	0.070	0.011
Objective orientation	0.050	0.020
Task accomplishment	0.066	-0.017
Cohesion	0.060	0.029
Willingness to accept responsibility	0.038	0.015
**Patients**		
decision-making/communication	0.148	0.108
self-management/ empowerment	0.105	0.050
psychosocial well-being	0.074	0.078

ICC: Intraclass Correlation Coefficient; t1: period of data collection before intervention; t2: period of data collection six months after intervention

### Results of staff survey

The means of teamwork variables were in a medium to positive range (for clinic-specific means, see Tables 5 and 6, for group-specific means, see Table 7). The comparison of baseline levels showed that means were higher for the control group than for the intervention group on all teamwork variables analyzed.

**Table 5:** Clinic specific means of outcome criteria of the staff survey (Cluster 1 to 3).

	Cluster 1 (Oncology)				Cluster 2 (Neurology)				Cluster 3 (Orthopaedics)			
	clinic 1 (IG)		clinic 7 (CG)		clinic 2 (IG)		clinic 8 (CG)		clinic 3 (IG)		clinic 9 (CG)	
	t1 (*n* = 15)	t2 (*n* = 13)	t1 (*n* = 40)	t2 (*n* = 32)	t1 (*n* = 40)	t2 (*n* = 32)	t1 (*n* = 39)	t2 (*n* = 19)	t1 (*n* = 29)	t2 (*n* = 14)	t1 (*n* = 32)	t2 (*n* = 23)
	*M (SD)*	*M (SD)*	*M (SD)*	*M (SD)*	*M (SD)*	*M (SD)*	*M (SD)*	*M (SD)*	*M (SD)*	*M (SD)*	*M (SD)*	*M (SD)*
Internal participation (IPS)	3.08 (0.58)	3.22 (0.46)	3.40 (0.54)	3.29 (0.54)	2.93 (0.57)	3.04 (0.63)	3.15 (0.34)	3.10 (0.44)	2.92 (0.26)	2.89 (0.23)	3.23 (0.34)	3.33 (0.54)
Team leadership (TeamPuls)	2.74 (0.69)	2.66 (0.40)	3.06 (0.44)	2.61 (0.44)	2.83 (0.66)	2.58 (0.57)	2.85 (0.64)	2.79 (0.54)	2.57 (0.51)	2.54 (0.70)	2.73 (0.64)	2.72 (0.54)
Team organization (TeamPuls)	2.71 (0.69)	2.80 (0.43)	3.10 (0.54)	2.78 (0.54)	2.73 (0.65)	2.78 (0.44)	2.86 (0.54)	2.84 (0.44)	2.97 (0.42)	3.04 (0.42)	2.99 (0.44)	2.95 (0.54)
Knowledge integration problems (WIP)	1.73 (1.06)	1.25 (0.76)	1.17 (0.74)	1.56 (1.04)	1.53 (0.73)	1.36 (0.78)	1.68 (0.54)	1.53 (0.84)	1.69 (0.49)	1.78 (0.44)	1.40 (0.74)	1.35 (0.94)
Objective orientation	4.81 (0.95)	4.98 (0.85)	5.16 (0.74)	4.83 (1.04)	4.78 (0.81)	4.61 (0.94)	4.58 (0.64)	4.87 (0.44)	4.83 (0.65)	4.82 (0.69)	5.02 (0.64)	4.91 (0.84)
Task accomplishment	3.83 (1.47)	4.73 (0.86)	4.81 (0.94)	4.56 (1.14)	4.07 (1.07)	4.47 (0.88)	4.09 (0.64)	4.59 (0.84)	4.56 (0.87)	4.46 (0.80)	4.52 (0.94)	4.56 (1.04)
Cohesion	3.89 (1.32)	4.50 (1.07)	4.55 (0.94)	4.12 (1.14)	4.59 (1.11)	4.68 (0.97)	4.18 (0.84)	4.48 (0.84)	4.08 (0.78)	3.92 (0.68)	4.32 (1.04)	4.31 (1.24)
Willingness to accept responsibility	3.72 (1.41)	4.12 (0.82)	4.68 (0.94)	3.97 (1.24)	4.18 (1.06)	4.43 (1.03)	4.15 (0.84)	4.26 (1.14)	4.10 (0.94)	4.07 (0.62)	4.30 (0.84)	4.36 (1.14)

t1: period of data collection before intervention; t2: period of data collection six months after intervention; M: mean; SD: standard deviation

**Table 6:** Clinic specific means of outcome criteria of the staff survey (Cluster 4 and 5).

	Cluster 4 (Orthopaedics/Cardiology)				Cluster 5 (Orthopaedics)			
	clinic 4 (IG)		clinic 6 (CG)		clinic 5 (IG)		clinic 10 (CG)	
	t1 (*n* = 30)	t2 (*n* = 23)	t1 (n=12)	t2 (n=14)	t1 (*n* = 10)	t2 (n=16)	t1 (n=14)	t2 (n=12)
	*M (SD)*	*M (SD)*	*M* (*SD*)	*M* (*SD*)	*M (SD)*	*M (SD)*	*M* (*SD*)	*M* (*SD*)
Internal participation (IPS)	3.02 (0.55)	3.09 (0.64)	3.51 (0.39)	3.17 (0.57)	3.27 (0.34)	3.38 (0.52)	3.30 (0.44)	3.31 (0.44)
Team leadership (TeamPuls)	2.51 (0.72)	2.57 (0.52)	3.04 (0.85)	2.63 (0.51)	2.90 (0.66)	2.77 (0.49)	2.93 (0.64)	2.91 (0.64)
Team organization (TeamPuls)	2.63 (0.54)	2.80 (0.37)	3.00 (0.66)	2.83 (0.43)	3.08 (0.44)	3.01 (0.39)	2.88 (0.44)	2.69 (0.44)
Knowledge integration problems (WIP)	1.74 (0.93)	1.25 (0.79)	1.00 (0.52)	1.19 (0.69)	1.30 (0.72)	1.28 (0.66)	1.06 (0.84)	1.11 (0.94)
Objective orientation	4.43 (1.07)	4.41 (1.05)	4.61 (1.17)	4.54 (1.05)	4.83 (0.68)	5.02 (0.72)	4.86 (0.74)	4.92 (0.84)
Task accomplishment	4.10 (1.22)	4.38 (1.16)	4.69 (1.03)	4.23 (0.96)	4.58 (0.68)	4.69 (0.95)	4.55 (0.84)	4.65 (1.04)
Cohesion	4.24 (1.14)	4.41 (1.09)	5.06 (0.54)	4.65 (0.85)	4.68 (0.73)	4.71 (0.91)	4.91 (0.74)	4.68 (1.14)
Willingness to accept responsibility	4.08 (1.13)	4.09 (1.08)	4.52 (0.98)	3.98 (1.16)	4.68 (0.73)	4.84 (0.72)	4.43 (0.94)	4.33 (1.14)

t1: period of data collection before intervention; t2: period of data collection six months after intervention; M: mean; SD: standard deviation

**Table 7:** Staff survey: Univariate comparisons of the patient orientation and teamwork variables for group and time of data collection.

	Intervention group		Control group		Variance analysis^1^					
	t1 *(n* = 141)	t2 *(n* = 113)	t1 *(n* = 164)	t2 *(n* = 100)	Main effect Group		Main effect Time		Interaction effect Group*Time	
	*M* (*SD*)	*M* (*SD*)	*M* (*SD*)	*M* (*SD*)	*p*	*η*^*2*^	*p*	*η*^*2*^	*p*	*η*^*2*^
Internal participation (IPS)	3.00 (0.50)	3.11 (0.56)	3.29 (0.43)	3.24 (0.52)	.000	.042	.504	.001	.093	.006
Team leadership (TeamPuls)	2.69 (0.65)	2.62 (0.54)	2.91 (0.63)	2.72 (0.55)	.005	.018	.029	.011	.342	.002
**Team organization (TeamPuls)**	**2.79 (0.58)**	**2.86 (0.42)**	**2.98 (0.52)**	**2.83 (0.49)**	.113	.006	.459	.001	**.027**	**.011**
Problems with knowledge integration (WIP)	1.62 (0.78)	1.37 (0.73)	1.34 (0.70)	1.38 (0.89)	.065	.008	.132	.005	.051	.009
Objective orientation	4.71 (0.86)	4.71 (0.91)	4.88 (0.74)	4.82 (0.86)	.078	.007	.686	.000	.745	.000
Task accomplishment	4.21 (1.11)	4.52 (0.94)	4.50 (0.89)	4.53 (1.00)	.116	.006	.073	.007	.138	.005
Cohesion	4.31 (1.07)	4.49 (0.98)	4.47 (0.92)	4.41 (1.06)	.654	.000	.535	.001	.221	.003
**Willingness to accept responsibility**	**4.12 (1.08)**	**4.32 (0.94)**	**4.40 (0.88)**	**4.19 (1.15)**	.455	.001	.977	.000	**.036**	**.010**

t1: period of data collection before intervention; t2: period of data collection six months after intervention; M: mean, SD: standard deviation; effect size: partial eta-square η^2^; η^2 = ^0.01 (small); η^2^=0.06 (medium); η^2^=0.14 (large)

^1^Pillai’s trace (multivariate test).

The multivariate analysis of teamwork variables showed significant main effects of time, *F*(8,434)=3.46, *p* < .01, *η*^2 = ^.060, and group, *F*(8,434)=4.03, *p* < .001 *η*^2 = ^.069, but no interaction effect between time and group, *F*(8,434)=1.54, *p=*.14, *η*^2 = ^.028. Subsequent univariate tests showed significant interaction effects for team organization and willingness to accept responsibility, with higher mean values for t2 than for t1 in the intervention group and higher mean values for t1 than for t2 in the control group (see Table 7).

Analyses excluding the staff of clinics 3 and 9 showed again significant main effects of time, *F*(8,336)=4.49, *p* < .001, *η*^2 = ^.097, and group, *F*(8,336)=2.50, *p* < .05 *η* ^2^=.056, and also a significant interaction effect between time and group, *F*(8,336)=2.18, *p* < .05, *η*^2 = ^.049. Subsequent univariate tests showed significant interaction effects for team organization, *F*(1,343)=6.01, *p* < .05, *η*^*2*^*=*.017, and willingness to accept responsibility, *F*(1,343)=5.68, *p* < .05, *η*^*2*^*=*.016, and additionally problems with knowledge integration, *F*(1,343)=4.39, *p* < .05, *η*^*2*^*=*.014, with higher mean values for t2 than for t1 in the intervention group and higher mean values for t1 than for t2 in the control group.

### Results of the patient survey

The comparison of baseline levels showed higher mean values for the control group than for the intervention group on all CCRQ scales.

The means of outcome criteria were in a medium to positive range (for clinic-specific means, see Tables 8 and 9, for group means see Table 10). The multivariate test showed a significant main effect of group, *F*(3,1028)=10.39, *p <* .001, *η*^2^=.029, whereas the main effect of time, *F*(3,1028)=1.81, *p*=.11, *η*^2 = ^.005, and the main effect of group x time, *F*(3,1028)=0.75, *p=*.52, *η*^*2*^*=*.002, were not significant. The tests of effects between subjects yielded a significant main effect of group for the CCRQ scales decision-making/communication, self-management/empowerment and psychosocial well-being, with higher means for the control than the intervention group (for univariate effects see Table 10).

**Table 8:** Clinic specific means of outcome criteria of the patient survey (Cluster 1 and 3).

	Cluster 1 (Oncology)				Cluster 3			
	clinic 1 (IG)		clinic 7 (CG)		clinic 3 (IG)		clinic 9 (CG)	
	t1 (*n* = 77)	t2 (*n* = 45)	t1 (*n* = 21)	t2 (*n* = 21)	t1 (*n* = 94)	t2 (*n* = 85)	t1 (*n* = 57)	t2 (*n* = 71)
	*M (SD)*	*M (SD)*	*M (SD)*	*M (SD)*	*M (SD)*	*M (SD)*	*M (SD)*	*M (SD)*
decision-making/communication	4.29 (0.73)	4.18 (0.75)	4.30 (0.67)	4.12 (0.63)	3.21 (0.97)	3.21 (1.13)	4.10 (0.84)	3.99 (0.82)
self-management/empowerment	3.93 (0.80)	3.78 (0.95)	3.95 (0.95)	3.81 (0.66)	3.07 (0.98)	3.09 (0.96)	3.87 (0.86)	3.75 (0.87)
psychosocial well-being	4.36 (0.69)	4.13 (0.90)	4.18 (0.96)	4.17 (0.80)	3.40 (0.96)	3.37 (1.11)	4.15 (0.87)	4.05 (0.83)

t1: period of data collection before intervention; t2: period of data collection six months after intervention; M: mean, SD: standard deviation

**Table 9:** Clinic specific means of outcome criteria of the patient survey (Cluster 4 and 5).

	Cluster 4 (Orthopaedics/Cardiology)				Cluster 5 (Orthopaedics)			
	clinic 4 (IG)		clinic 6 (CG)		clinic 5 (IG)		clinic 10 (CG)	
	t1 (*n* = 68)	t2 (*n* = 101)	t1 (*n* = 48)	t2 (*n* = 51)	t1 (*n* = 38)	t2 (*n* = 77)	t1 (*n* = 40)	t2 (*n* = 15)
	*M (SD)*	*M (SD)*	*M (SD)*	*M (SD)*	*M (SD)*	*M (SD)*	*M (SD)*	*M (SD)*
decision-making/communication	3.63 (0.99)	3.90 (0.75)	4.20 (0.67)	3.81 (0.90)	3.77 (0.91)	3.83 (0.87)	3.74 (1.10)	4.00 (0.71)
self-management/empowerment	3.43 (0.92)	3.55 (0.87)	4.05 (0.75)	3.68 (0.93)	3.60 (0.91)	3.55 (0.91)	3.44 (1.18)	3.76 (0.77)
psychosocial well-being	3.85 (0.81)	3.94 (0.84)	4.26 (0.73)	3.76 (0.89)	4.11 (0.61)	3.99 (0.75)	3.87 (0.93)	4.03 (0.71)

t1: period of data collection before intervention; t2: period of data collection six months after intervention, M: mean; SD: standard deviation

**Table 10:** Patient survey: Univariate comparisons of CCRQ scales for group and time of data collection.

	Intervention group		Control group		Variance analysis^1^					
	t1 *(n* = 351)	t2 *(n = *381)	t1 *(n* = 172)	t2 *(n* = 164)	Main effect Group		Main effect Time		Interaction effect Group*Time	
	*M* (*SD*)	*M* (*SD*)	*M* (*SD*)	*M* (*SD*)	*p*	*η*^*2*^	*p*	*η*^*2*^	*p*	*η*^*2*^
decision-making/communication	3.70 (1.00)	3.76 (0.95)	4.07 (0.87)	3.95 (0.81)	.000	.019	.663	.000	.164	.002
self-management/empowerment	3.45 (1.00)	3.48 (0.96)	3.83 (0.95)	3.73 (0.85)	.000	.023	.660	.000	.318	.001
psychosocial well-being	3.95 (0.89)	3.87 (0.93)	4.12 (0.86)	3.97 (0.84)	.026	.005	.051	.004	.570	.000

t1: period of data collection before intervention; t2: period of data collection six months after intervention; M: mean; SD: standard deviation; effect size: partial eta-square=*η*^2^; *η*^2 = ^0.01 (small); *η*^2^=0.06 (medium); *η*^2^=0.14 (large)

^1^Pillai’s trace (multivariate test).

As in the staff survey, the analysis was conducted again excluding patients of clinics 3 and 9 (and also clinic 8 for the patient survey only).

The multivariate test showed a significant main effect of group, *F*(3,721)=3.77, *p=*.01, *η*^2 = ^.015, although the main effect of time, *F*(3,721)=1.51, *p=*.21, *η*^2^=.006, and the main effect of group x time, *F*(3,721)=0.48, *p=*.70, *η*^*2*^*=*.002, were not significant. The tests of effects between subjects yielded a significant main effect of group for the CCRQ scale self-management/empowerment, *F*(3,723)=4.57, *p <* .05, *η*^2 = ^.006, with higher mean values for the control group (*M=*3.77, *SD=*0.07) than for the intervention group (*M=*3.60, *SD=*0.04).

## Discussion

Overall, for some dimensions of teamwork, there were small significant interaction effects between the intervention and control group over time in the staff survey. Analysis showed that after the intervention means of dimension such as team organization and willingness to accept responsibility (and knowledge integration when excluding two clinics from the analysis) improved. Those univariate effects must be regarded as small [42]. The multivariate interaction effect over all analyzed teamwork dimensions in the staff survey only reached significance when excluding two clinics from analysis and could then be considered as moderate, with the restriction that this result can only be regarded a hint towards possible additional effects if staff support TCC. Descriptive statistics showed that effects were due to an improvement in the intervention group and a decline in mean values in the control group. For other teamwork-related processes (e.g., objective orientation, task accomplishment, cohesion), the intervention did not result in significant improvements. Therefore, hypothesis one, that the TCC will improve interprofessional teamwork in medical rehabilitation, was partly supported for some dimensions of teamwork. Hypothesis two, which states that the team intervention concept can enhance the external participation aspect of patient-centeredness, could not be confirmed by the results of the patient survey.

The effects on specific teamwork dimensions such as organization, responsibility and knowledge integration can be explained by looking at the main themes addressed by the TCC and by the requests of team members and executives expressed in the pilot study [31]. Although requests varied among clinics, some common themes could be identified, such as an optimization of team meetings [44]. Consistent with the literature [45], improvements in the organization of team meetings, optimal knowledge and information exchange about patients and agreements on responsibilities were the focus of the interviews and focus groups in the pilot study.

The rather small effect sizes in our study may be related to the fact that all employees in the clinics were surveyed, rather than only those who participated in the team intervention. This approach was deliberately chosen because employees are often members of multiple teams, meaning that dissemination processes in the sense of organizational learning may be initiated. Even so, the TCC has initiated some processes of change, such as improvements in team organization and knowledge integration that can be regarded as a basis for other, slower processes. However, such transfer processes take time, and the intervention may also have been too specific to be able to involve greater changes in the whole organization.

Given the fact that only a small part of staff completing the questionnaires actually took part in the team training (56 of 230 at t2), it would be interesting to have a subgroup analysis of only those staff members that participated in the training. Regrettably, few staff members filled in the code that would allow for matched comparisons, and many staff members took the survey only once. Therefore the sample of staff that took part in the intervention and traceably completed two questionnaires is too small to calculate the inferential statistics used in this study.

In line with the small effect sizes in the staff survey, the missing effects in the patient survey can be explained by the fact that although the approach was patient-centered, the team intervention only targeted staff; there was no intervention in which patients themselves could participate. A combined intervention that includes information materials, decision-making support and patient education units would probably be perceived as more effective for improving patient-centeredness. Other studies in the medical setting have shown combined interventions to be effective in enhancing patient-centered care [46–48]. Moreover, it is very likely that different patient populations were asked to participate during the two data collection periods. Even though the samples were comparable, there could be individual differences between these two samples, for instance in terms of situational awareness and expectations. Another possibility is that the absent patient effect is due to the small staff effect, meaning that changes or improvements might have been too small or too specific to be recognizable by patients or that it would require more time for patients to notice effects.

Although common themes regarding the needs for team training could be identified (see also [31]), the team intervention was need-specific at a clinic level. Hence, the contents of the clinic-specific interventions were not standardized, although the process of the intervention was. As a result, reproducibility between clinics can be considered limited. However, a description of the concept can be found in a manual that gives practitioners guidelines and toolkits for carrying out a team intervention based on the principles developed in our study [33]. There might also be unknown selection effects both on a clinic and individual level. Due to the fact that participation in the study was voluntary, we do not know if only those clinics took part that are especially open to measures for improving the quality of treatment and as a result already practice better teamwork and patient-centered care or if particularly clinics with a high demand for team development and thus a lower level of teamwork and patient-centeredness might have taken part. This is, however, a natural self-selection process for interventions, with only those taking part who are motivated for one reason or another. This goes along with the fact that the TCC only targets clinics that see a need for improvement, and it would not be recommended to “convince” clinics to take part in an intervention. Nevertheless, it would be interesting to examine if the TCC is only effective under special conditions and why some clinics were not interested in the TCC. This should be part of future research. Certainly, one reason might be the time required to participate in an intervention during routine operations on the ward, and it would be of interest to find ways to motivate clinics and design interventions in a way that they seem applicable to a broad range of clinics. Limitations can also be found in the data analysis. Although the intervention and control clinics were assigned randomly, the data analysis showed that the baseline levels for the outcome criteria diverged significantly, with better baseline levels in the control clinics both in the staff and the patient survey. Moreover, the deterioration of means in the control group over time suggests a different explanation. Employees who were dissatisfied with teamwork at their clinics may have been more likely to complete the quite extensive questionnaire for a second time, whereas employees who were satisfied may have been less motivated to complete it again. Another limiting factor is the low, but not unusual, response rate. Since drop-out analysis was not possible, an attrition bias might exist. However, the process evaluation [43] showed that staff accepted the training, so that we cannot draw the conclusion that the low response rate was due to low engagement or acceptance; instead, it is probably due to the high workload of staff. On the other hand it must be noted that of 71 participants in the training, 56 completed the questionnaire at t2. Here it should also be acknowledged that one clinic failed to engage with the intervention because of persisting conflict on a more global, structural level. This shows that the intervention is not suitable for solving problems that go beyond the team level.

Regarding the statistical analysis, as mentioned above, a MANOVA is not the optimal statistical procedure to examine the research question, which has to be regarded as a limitation of the study. Unfortunately, the data did not allow calculating a repeated measures design as only very few of the participants filled in the code that allows matching the questionnaires.

In summary, the TCC can be recommended to improve teamwork, especially team organization, willingness to accept responsibility and knowledge integration. The TCC meets the challenges of a holistic treatment approach by optimizing knowledge integration of the different health care professionals working together in an interprofessional team. The TCC is a combination of focusing tasks, processes and cooperation in the team. It supported teams in their reflection how to accomplish the common task best. It is a time-saving and effective approach to both use the capabilities of every team member and join together to become a whole team.

The first implementation showed that the concept is well accepted by the teams and is a feasible team development approach. As the first team intervention approach for rehabilitation clinics in Germany, it permits a standardized procedure but since every team is unique it is needs-specific and therefore applicable to different clinical settings where effective teamwork is required. The approach has been evaluated in a cluster-randomized controlled study and, as one of very few studies, also considered the patient perspective in its development [4]. A further evaluation of the approach should be carried out in a larger study that includes more clinics. Furthermore, collecting data at more points in time would both allow for a continuous formative evaluation and help to measure processes that might take longer than six months. The collection of qualitative data could help answer unresolved questions regarding how the intervention was perceived by staff and what factors potentially lead to success or failure of an intervention. It is suspected that there might be effects of the intervention that were not captured by the assessment tools, such as effects on information flow or the effectiveness of team meetings. Those gaps are estimated to be filled in a follow-up study with a more qualitative design. In further a study, multilevel analyses might also bring to light structural conditions on the clinic level that benefit or hinder the implementation of the intervention.

To achieve sustainable improvements in healthcare, the TCC is manualized, and a train-the-trainer concept will be developed on its basis in order to achieve more widespread use of the approach in the future. The aim is to empower team leaders to coach their teams rather than employ an external counselor. Furthermore, the TCC is not specific to rehabilitation. It could also be used in acute care or other health care settings because content can be matched individually.

## Supporting information

S1 SPSS SPSS datafile of staff data. Data of staff survey for both data collection periods without missing values.
(SAV)

S2 SPSS SPSS datafile of patient data. Data of patient survey for both data collection periods without missing values.
(SAV)

S1 Quest Staff questionnaire. (PDF)

S2 Quest Patient questionnaire. (PDF)
