# Correction: A cluster-randomized controlled study to evaluate a team coaching concept for improving teamwork and patient-centeredness in rehabilitation teams

**DOI:** 10.1371/journal.pone.0190338

**Published:** 2017-12-21

**Authors:** Mirjam Körner, Leonie Luzay, Anne Plewnia, Sonja Becker, Manfred Rundel, Linda Zimmermann, Christian Müller

The following information is missing from the Funding section: The article processing charge was funded by the German Research Foundation (DFG) and the University of Freiburg in the funding programme Open Access Publishing.
