# Supplementary material for: A cluster-randomized controlled study to evaluate a team coaching concept for improving teamwork and patient-centeredness in rehabilitation teams
Source: PLoS One. 2017 Jul 13;12(7):e0180171. doi: 10.1371/journal.pone.0180171 (PMC5509115; doi:10.1371/journal.pone.0180171)
Supplement: S1 Quest — (PDF) [file pone.0180171.s003.pdf]

Wie sollen die Fragen ausgefüllt werden?
Bitte kreuzen Sie jeweils **nur eine Antwortmöglichkeit pro Frage** an, wenn nichts anderes vorgegeben ist. Für uns ist es wichtig, dass Sie spontan alle Fragen beantworten. Wir möchten in diesem Zusammenhang darauf hinweisen, dass die Kreuze immer in ein vorgesehenes Kästchen zu setzen sind und bitten Sie, nicht zwischen den Bewertungsmöglichkeiten anzukreuzen. Es gibt bei keiner Frage richtige oder falsche Antworten. Entscheidend für Ihre Antworten sind allein **Ihre persönlichen Erfahrungen und Meinungen**.

Ihr persönlicher Code?
Damit wir bei späteren – ebenfalls anonymen – Befragungen Ihren Fragebogen richtig zuordnen können, bitten wir Sie um die Erstellung eines persönlichen Codes, den nur Sie kennen. Bitte benützen Sie dabei die folgende Regel:

Zweiter Buchstabe des **Vornamens** Ihrer leiblichen **Mutter**,

zweiter Buchstabe des **Geburtsnamens** (Mädchenname) Ihrer leiblichen **Mutter**,

dritter Buchstabe des **Geburtsnamens** (Mädchenname) Ihrer leiblichen **Mutter**,

zweiter Buchstabe Ihres eigenen **Geburtsortes**,

Geburtsjahr Ihrer leiblichen **Mutter.**

FAT-Scale

B. Die folgenden Aussagen dienen der umfassenden Beschreibung **der Zusammenarbeit Ihres interprofessionellen Reha-Teams**. Bitte geben Sie an, inwieweit die folgenden Aussagen zutreffen.

1.	Die Ziele unseres Teams sind uns klar.	○ ○ ○ ○ ○ ○	Uns sind die Ziele des Teams unklar.
2.	Alle bringen sich in gleichem Maße in das Team ein.	○ ○ ○ ○ ○ ○	Einige lassen sich von den anderen Teammitgliedern durchziehen.
3.	Unsere Ziele sind realistisch und erreichbar.	○ ○ ○ ○ ○ ○	Unsere Ziele sind unrealistisch und unerreichbar.
4.	Unsere Prioritäten sind unklar.	○ ○ ○ ○ ○ ○	Unsere Prioritäten sind klar.
5.	Wir erreichen alle Ziele mit Leichtigkeit.	○ ○ ○ ○ ○ ○	Manchmal haben wir den Eindruck, dass wir die Ziele nicht erreichen.
6.	Die Teammitglieder kennen ihre Aufgaben.	○ ○ ○ ○ ○ ○	Die Teammitglieder wissen nicht genau, was sie zu tun haben.
7.	Wir koordinieren unsere Anstrengungen schlecht.	○ ○ ○ ○ ○ ○	Wir koordinieren unsere Anstrengungen gut.
8.	Wir reden offen und frei miteinander.	○ ○ ○ ○ ○ ○	Wir reden nicht offen und frei miteinander.
9.	Wir bringen alle wichtigen Informationen in unser Team ein.	○ ○ ○ ○ ○ ○	Wir behalten Informationen für uns.

10. Wir fühlen uns untereinander verstanden und akzeptiert.	○ ○ ○ ○ ○ ○	Wir fühlen uns untereinander unverstanden und nicht akzeptiert.
11. Einige denken zuviel an sich selbst.	○ ○ ○ ○ ○ ○	Das Team steht im Mittelpunkt und nicht der Einzelne.
12. Es gibt niemals Spannungen im Team.	○ ○ ○ ○ ○ ○	Manchmal gibt es Spannungen unter den Teammitgliedern.
13. Es gibt Konkurrenz zwischen den Teammitgliedern.	○ ○ ○ ○ ○ ○	Konkurrenz zwischen den Teammitgliedern ist kein Thema.
14. Die Teammitglieder vermeiden es, Verantwortung zu übernehmen.	○ ○ ○ ○ ○ ○	Die Mitglieder übernehmen Verantwortung.
15. Die Anforderungen an unsere Arbeitsergebnisse sind klar formuliert.	○ ○ ○ ○ ○ ○	Die Anforderungen an unsere Arbeitsergebnisse sind nicht klar formuliert.
16. Die Teammitglieder helfen sich gegenseitig, wenn einer in Zeitnot gerät.	○ ○ ○ ○ ○ ○	Die Teammitglieder helfen sich nicht, wenn einer in Zeitnot gerät.
17. Einzelne Mitglieder versuchen sich – auf Kosten anderer – in den Vordergrund zu drängen.	○ ○ ○ ○ ○ ○	Es versucht niemand, sich – auf Kosten anderer – in den Vordergrund zu drängen.
18. Wir denken ständig über Verbesserungen nach.	○ ○ ○ ○ ○ ○	Wir denken selten über Verbesserungen nach.
19. Ich identifiziere mich mit den Zielen des Teams.	○ ○ ○ ○ ○ ○	Ich identifiziere mich nicht mit den Zielen des Teams.
20. Informationen werden rechtzeitig ausgetauscht.	○ ○ ○ ○ ○ ○	Informationen werden oft zu spät ausgetauscht.
21. Die Erreichung unserer Ziele ist wichtig für die Gesamtorganisation.	○ ○ ○ ○ ○ ○	Die Erreichung unserer Ziele ist unwichtig für die Gesamtorganisation.
22. Wir haben Kriterien, um den Grad der Zielerreichung bestimmen zu können.	○ ○ ○ ○ ○ ○	Wir haben keine Kriterien, um den Grad der Zielerreichung bestimmen zu können.
23. In unserem Team fühlt sich jeder für das Gesamtergebnis der Arbeit verantwortlich.	○ ○ ○ ○ ○ ○	In unserem Team fühlt sich nicht jeder für das Ergebnis verantwortlich.
24. Wir fühlen uns als ein Team.	○ ○ ○ ○ ○ ○	Ein „Wir-Gefühl“ ist in unserem Team unterentwickelt.

**Gemeinsames Aufgabenverständnis**	trifft nicht zu	trifft eher nicht zu	trifft manchmal zu	trifft eher zu	trifft völlig zu
25. Teammitglieder können die eigene Sichtweise den anderen Teilnehmern nur unzureichend verständlich machen.	⓪	①	②	③	④
26. Es wird zu wenig Zeit und Mühe aufgewendet, um ein gemeinsames Aufgabenverständnis zu entwickeln.	⓪	①	②	③	④
27. Teammitglieder bemühen sich zu wenig, die Denkweisen und methodischen Vorgehensweisen der anderen Fachrichtungen zu verstehen.	⓪	①	②	③	④
28. Teammitglieder haben unterschiedliche Zielvorstellungen für die Erledigung ihrer Aufgabe.	⓪	①	②	③	④
29. Die interprofessionelle Zusammensetzung des Teams erschwert die Entwicklung eines gemeinsamen Aufgabenverständnisses.	⓪	①	②	③	④
30. Die fachspezifischen Denkweisen der einzelnen Teammitglieder erschweren die Bildung einer gemeinsamen Basis für die Erledigung der Aufgabe.	⓪	①	②	③	④
31. Die Methoden der anderen Fachrichtungen bleiben unklar.	⓪	①	②	③	④

KIP Scale; in German: WIP-Skala

Teamskala	trifft überhaupt nicht zu	trifft eher nicht zu	trifft eher zu	trifft voll und ganz zu	kann ich nicht beurteilen
32. Die Teammitglieder arbeiten Hand in Hand.	①	②	③	④	○
33. Die Kommunikation im Team funktioniert gut.	①	②	③	④	○
34. Absprachen zwischen den Teammitgliedern untereinander funktionieren gut.	①	②	③	④	○
35. In der Klinik herrscht insgesamt ein freundliches Klima.	①	②	③	④	○
36. Die einzelnen Teammitglieder gehen respektvoll miteinander um.	①	②	③	④	○
37. Die einzelnen Behandlungen sind gut aufeinander abgestimmt.	①	②	③	④	○

IPS-Scale

	ungenügend	mangelhaft	ausreichend	befriedigend	gut	sehr gut
38. Insgesamt bewerte ich die interprofessionelle **Teamarbeit** in meiner Klinik als …	○	○	○	○	○	○
39. Insgesamt bewerte ich die interprofessionelle **Teamleistung** in meiner Klinik als …	○	○	○	○	○	○

A. Die folgenden Aussagen beziehen sich auf die **Führung des interprofessionellen Reha-Teams** und die **Teamorganisation**. Bitte geben Sie an, inwieweit die folgenden Aussagen zutreffen.

1. Es ist eine Teamleitung benannt. ○ ja ○ nein

Aussagen zur Teamführung	trifft gar nicht zu	trifft weniger zu	trifft eher zu	trifft völlig zu
2. Der Teamleiter geht differenziert auf Fähigkeiten und Engagement der einzelnen Teammitglieder ein.	①	②	③	④
3. Die Teammitglieder werden über alle Belange, die das Team betreffen, vom Teamleiter regelmäßig auf dem Laufenden gehalten.	①	②	③	④

Team-Puls Scale

	trifft gar nicht zu	trifft weniger zu	trifft eher zu	trifft völlig zu
4. Der Teamleiter vertritt die Interessen des Teams zur vollen Zufriedenheit der Teammitglieder wirksam nach außen.	①	②	③	④
5. Der Teamleiter versucht gelegentlich, eigene Versäumnisse auf andere im Team abzuwälzen.	④	③	②	①
6. Bei der Rückmeldung durch den Teamleiter stehen Anerkennung und Kritik in einem ausgewogenen Verhältnis.	①	②	③	④
7. Konflikte im Team werden vom Teamleiter direkt angesprochen und nicht dem Selbstlauf überlassen.	①	②	③	④
8. Der Teamleiter fördert in vorbildlicher Weise den Zusammenhalt im Team.	①	②	③	④
9. Der Teamleiter trifft wichtige Entscheidungen, von denen Teammitglieder betroffen sind, *nicht* im Alleingang.	①	②	③	④
**Aussagen zur Teamorganisation**				
10. Teambesprechungen werden sorgfältig vorbereitet. Inhalte, Ziele und Ablauf sind klar.	①	②	③	④
11. Die Vertretung von Teammitgliedern, z. B. bei Urlaub und Krankheit, ist eindeutig geklärt.	①	②	③	④

		①	②	③	④
12.	Der Informationsaustausch zwischen den einzelnen Teammitgliedern ist so geregelt, dass eine optimale Aufgabenerledigung möglich ist.	①	②	③	④
13.	Das Team sichert eine gute Arbeitsqualität durch Einbindung von Fachexperten und gegenseitige Kontrolle.	①	②	③	④
14.	Die Aufgaben der einzelnen Teammitglieder sind eindeutig geklärt. Jeder weiß genau, was er zu tun hat.	①	②	③	④
15.	Alle kennen neben ihren eigenen Aufgaben auch die der anderen Teammitglieder. Die Schnittstellen sind geklärt.	①	②	③	④
16.	Bevor sich das Team für eine Lösung entscheidet, werden erst mehrere Alternativen gegeneinander abgewogen	①	②	③	④
17.	Die gemeinsame Arbeit wird manchmal nicht genügend koordiniert und geplant.	④	③	②	①

		überhaupt nicht wichtig	eher unwichtig	eher wichtig	sehr wichtig
18.	Wie wichtig finden Sie das Thema „patientenorientierte Teamentwicklung“ für Ihre Klinik?	①	②	③	④

E. Die folgenden Fragen beziehen sich auf **Ihre Person** und **Ihren Beruf**. Bitte kreuzen Sie das auf Sie Zutreffende an. Diese Angaben benötigen wir, um zu überprüfen, ob die Studienteilnehmer in unterschiedlichen Reha-Einrichtungen insgesamt vergleichbar sind.

Socio-demo-graphy

1. Welcher Berufsgruppe gehören Sie an?

① Arzt
② Pflegepersonal
③ Physio-, Sporttherapeut, Sportlehrer, Masseur, Bademeister
④ Psychologe, Psychotherapeut
⑤ Sozialpädagoge, Sozialarbeiter
⑥ Ernährungsberater, Diätassistent
⑦ Logopäde
⑧ Ergotherapeut
⑨ Kunst-, Musik-, Gestaltungstherapeut
⑩ Sonstige: ________________________

2. Haben Sie eine leitende Position in Ihrer Einrichtung?

○ nein ○ ja, mittlere Führungsebene ○ ja, obere Führungsebene

3. Wie lange sind Sie in dieser Klinik schon tätig?

① 0 bis 5 Jahre
② 6 bis 10 Jahre
③ 11 bis 15 Jahre
④ länger als 15 Jahre

4. Wie alt sind Sie?

○ unter 30 Jahre ○ 30 bis 39 Jahre ○ 40 bis 49 Jahre ○ 50 bis 59 Jahre ○ älter als 59 Jahre

5. Welches Geschlecht haben Sie? ○ weiblich ○ männlich

6. Wie viel Prozent arbeiten Sie?

① weniger als 50%
③ mehr als 75%, aber weniger als 100%
② zwischen 50% und 75%
④ 100%

7. Falls Sie der Ansicht sind, dass wir bei dieser Befragung einen wichtigen Aspekt vergessen haben oder Sie sonstige Anregungen oder Kritik haben, finden Sie hier Platz, um uns dies mitzuteilen.

Bitte Rückseite beachten!

H. Die folgenden Fragen beziehen sich auf Ihre **Teilnahme an den einzelnen Sitzungen der Teamentwicklung in ihrer Klinik**. Bitte beantworten Sie bei den nachfolgenden Fragen, inwiefern Sie als Mitarbeiter im Rahmen des Projektes PATENT an den einzelnen Sitzungen der Teamentwicklung beteiligt waren. Bitte kreuzen Sie das auf Sie Zutreffende an.

H1. Haben Sie im Rahmen des Projektes PATENT an einer oder mehreren Sitzungen der Teamentwicklung teil genommen?

① Nein (Wenn nein: weiter bei Frage H3.) ② Ja

H2. An wie vielen Terminen haben Sie teil genommen?

① Ich habe an allen Terminen teilgenommen

② Ich habe 1 mal teilgenommen

③ Ich habe 1-2 mal teilgenommen

④ Ich habe 3-5 mal teilgenommen

H3. Gab es in Ihrer Klinik im letzen Jahr anderweitige Team-/ oder Organisationsberatungen?

① Nein ② Ja

I. Die nachfolgende Frage bezieht sich auf **Ihren Informationsstand über das Projekt „Patientenorientierte Team-Entwicklung (PATENT)"**. Bitte kreuzen Sie das auf Sie Zutreffende an.

I1. Wurden Sie in Ihrer Klinik über das Projekt „Patientenorientierte Team-Entwicklung (PATENT)" informiert?

① Nein ② Ja (Wenn ja: weiter bei I2.)

I2. Von wem haben Sie die Informationen über das Projekt „Patientenorientierte Team-Entwicklung (PATENT)" erhalten?

① Ich wurde über die Abteilungsleitung darüber informiert.

② Ich habe im Austausch mit Kollegen darüber erfahren.

③ Ich wurde von der Klinikleitung darüber informiert.

④ Ich habe über einen anderen Weg davon erfahren ____________________________ .

Herzlichen Dank!
