# Supplementary material for: A cluster-randomized controlled study to evaluate a team coaching concept for improving teamwork and patient-centeredness in rehabilitation teams
Source: PLoS One. 2017 Jul 13;12(7):e0180171. doi: 10.1371/journal.pone.0180171 (PMC5509115; doi:10.1371/journal.pone.0180171)
Supplement: S2 Quest — (PDF) [file pone.0180171.s004.pdf]

C

Die folgenden Fragen beziehen sich auf Ihre Erfahrungen als Patient während Ihres Rehabilitationsaufenthaltes. Mit dem Begriff „Behandlungsteam“ sind alle Berufsgruppen gemeint, die an Ihrer Behandlung beteiligt sind. Kreuzen Sie bitte jeweils an, inwieweit Sie den folgenden Aussagen zustimmen.

CCRQ

	stimme überhaupt nicht zu	stimme nicht zu	weder noch	stimme zu	stimme voll zu	kann ich nicht beurteilen
1. Das Behandlungsteam und ich entscheiden gemeinsam darüber, was mir helfen würde.	①	②	③	④	⑤	⓪
2. Ich hatte Schwierigkeiten, die Informationen zu meiner Gesundheit zu bekommen, die ich benötigte.	①	②	③	④	⑤	⓪
	stimme überhaupt nicht zu	stimme nicht zu	weder noch	stimme zu	stimme voll zu	kann ich nicht beurteilen
3. Ich war stets gut informiert über meine Fort-schritte in den für mich wichtigen Bereichen.	①	②	③	④	⑤	⓪
4. Meine Familie/Freunde bekamen die für sie notwendige Unterstützung vom Behandlungsteam.	①	②	③	④	⑤	⓪
5. Das Behandlungsteam behandelte mich als Mensch und nicht nur als irgendeinen Fall.	①	②	③	④	⑤	⓪
6. Das Behandlungsteam versuchte, meine Bedürfnisse bei der Terminplanung zu berücksichtigen.	①	②	③	④	⑤	⓪
7. Ich musste für die verschiedenen Behandler alle Informationen wiederholen.	①	②	③	④	⑤	⓪
8. Meine körperlichen Schmerzen wurden so gut wie möglich kontrolliert.	①	②	③	④	⑤	⓪
9. Das Behandlungsteam berücksichtigt meine persönlichen Bedürfnisse bei der Behandlungsplanung.	①	②	③	④	⑤	⓪
10. Ich bekam ausreichend Informationen über örtliche Unterstützungsangebote.	①	②	③	④	⑤	⓪

11. Ich habe in der Rehabilitation das geschafft, was ich mir vorgenommen habe.	①	②	③	④	⑤	⓪
12. Meine Familie/Freunde bekamen die gewünschte Information, wenn sie diese benötigten.	①	②	③	④	⑤	⓪
13. Ich wurde mit Respekt und Würde behandelt.	①	②	③	④	⑤	⓪
14. Meine Äußerungen zu meinen Schmerzen wurden vom Behandlungsteam ernst genommen	①	②	③	④	⑤	⓪
15. Meine Behandlungsbedürfnisse, Prioritäten und Ziele waren dem Behandlungsteam wichtig.	①	②	③	④	⑤	⓪
	stimme überhaupt nicht zu	stimme nicht zu	weder noch	stimme zu	stimme voll zu	kann ich nicht beurteilen
16. Das Behandlungsteam und ich diskutierten gemeinsam meine Fortschritte und passten, wenn notwendig die Behandlung an.	①	②	③	④	⑤	⓪
17. Meine Familie/Freunde bekamen Informationen, wie sie meine Versorgung Zuhause unterstützen können.	①	②	③	④	⑤	⓪
18. Ich wusste, an wen ich mich bei Problemen und Fragen während der Rehabilitation wenden konnte.	①	②	③	④	⑤	⓪
19. Ich hatte ausreichend Zeit, mich auszuruhen und zu schlafen.	①	②	③	④	⑤	⓪
20. Ich wurde ermutigt, mich bei der Zielsetzung zu beteiligen.	①	②	③	④	⑤	⓪
21. Ich bekam die notwendigen Informationen, wenn ich sie wollte.	①	②	③	④	⑤	⓪
22. Ich lernte, was ich wissen musste, um meine Krankheit Zuhause zu meistern.	①	②	③	④	⑤	⓪
23. Meine Familie/Freunde wurden mit Respekt behandelt.	①	②	③	④	⑤	⓪

Bitte Rückseite beachten!

24. Ich wusste, an wen ich mich bei Problemen nach der Entlassung wenden konnte.	①	②	③	④	⑤	⓪
25. Die Behandlungsoptionen wurden mir vollständig erklärt.	①	②	③	④	⑤	⓪
26. Meine Behandlung wurde mir verständlich erklärt.	①	②	③	④	⑤	⓪
27. Meine Familie/Freunde wurden in dem Ausmaß, wie ich wollte, in meine Rehabilitation einbezogen.	①	②	③	④	⑤	⓪
28. Ich konnte meine Gefühle gegenüber dem Behandlungsteam frei äußern.	①	②	③	④	⑤	⓪
29. Mir wurde gesagt, was ich zu erwarten hatte, wenn ich nach Hause kam.	①	②	③	④	⑤	⓪
	stimme überhaupt nicht zu	stimme nicht zu	weder noch	stimme zu	stimme voll zu	kann ich nicht beurteilen
30. Das Behandlungsteam bemühte sich um mein Wohlbefinden.	①	②	③	④	⑤	⓪
31. Meine emotionalen Bedürfnisse, Befürchtungen wie auch Ängste wurden von Seiten des Behandlungsteams ernst genommen und auf diese wurde eingegangen.	①	②	③	④	⑤	⓪
32. Meine Therapeuten, Pflegekräfte und Ärzte arbeiteten gut zusammen.	①	②	③	④	⑤	⓪
33. Es gab Zeiten, in denen ich mehr Informationen bekam, als ich aufnehmen konnte.	①	②	③	④	⑤	⓪
34. Bei Entscheidungen bezüglich meiner Behandlung wurde ich mit einbezogen.	①	②	③	④	⑤	⓪
35. Die Klinik ist auf den Patienten und dessen Bedürfnisse ausgerichtet.	①	②	③	④	⑤	⓪

F

Die folgenden Fragen beziehen sich auf Ihre Person und Ihren Beruf. Bitte kreuzen Sie das auf Sie Zutreffende an.

Angaben zur Person

1. Wie alt sind Sie?

[   ] Jahre

2. Welches Geschlecht haben Sie?

① weiblich ② männlich

3. Wie ist Ihr Familienstand?

① ledig
② geschieden / getrennt lebend
③ verheiratet
④ verwitwet

4. Sind Sie erwerbstätig?

○ **Ja,** ich bin erwerbstätig .... →
① weniger als 50%
② zwischen 50% und 75%
③ mehr als 75%, aber weniger als 100%
④ 100%

○ **Nein**, ich bin nicht erwerbstätig .... →
⑤ in Ausbildung oder Umschulung
⑥ arbeitslos gemeldet/ erwerbslos
⑦ Erwerbs-, Berufsunfähigkeitsrente
⑧ im vorzeitigen Ruhestand
⑨ ganz in Altersrente/pensioniert
⑩ Sonstiges / Anderes ______________

5. Wegen welcher Art von Erkrankung befinden Sie sich derzeit in Rehabilitation?

① orthopädische/rheumatologische
② kardiologische
③ neurologische
④ onkologische
⑤ psychosomatische/Sucht
⑥ sonstige: ____________________

6. Waren Sie vorher schon mal zur Rehabilitation?

① nein ② ja .... → Falls ja, wie oft? ___ mal

7. Welchen Schulabschluss haben Sie?

① keinen Schulabschluss
② Hauptschule / Volksschule
③ Realschule / Mittlere Reife
④ Fachhochschulreife
⑤ Abitur / allgemeine Hochschulreife
⑥ anderen Schulabschluss

8. Zu welchem Zeitpunkt sind die gesundheitlichen Einschränkungen, die Anlass für diese Rehabilitationsmaßnahme waren, zum ersten Mal aufgetreten?

① vor weniger als 3 Monaten
② vor etwa 3 bis 6 Monaten
③ vor etwa 6 bis 12 Monaten
④ vor etwa 1 bis 3 Jahren
⑤ vor etwa 3 bis 6 Jahren
⑥ vor mehr als 6 Jahren

9. In welcher beruflichen Stellung sind Sie derzeit hauptsächlich beschäftigt. Falls Sie nicht mehr berufstätig sind, in welchem Beschäftigungsverhältnis waren Sie zuletzt beschäftigt?

① Arbeiter
② Selbständiger
③ Angestellter
④ Beamter
⑤ Sonstiges ________________

Falls Sie der Ansicht sind, dass wir bei dieser Befragung einen wichtigen Aspekt vergessen haben oder Sie sonstige Anregungen oder Kritik haben, finden Sie hier Platz, um uns dies mitzuteilen.

____
____
____
____
____
____
____
____

Vielen Dank für die Beantwortung der Fragen!
